# Growth Differentiation Factor 15 and Matrix Metalloproteinase 3 in Plasma as Biomarkers for Neuropathy and Nephropathy in Type 1 Diabetes

**DOI:** 10.3390/ijms25137328

**Published:** 2024-07-03

**Authors:** Karin Billeson, Evangelia Baldimtsi, Jeanette Wahlberg, Per A. Whiss

**Affiliations:** 1 Department of Biomedical and Clinical Sciences, Division of Clinical Chemistry and Pharmacology, Linköping University, 581 83 Linköping, Sweden; karbi060@student.liu.se; 2 Department of Acute Internal Medicine and Geriatrics in Linköping, Department of Health, Medicine and Caring Sciences, Linköping University, 581 83 Linköping, Sweden; evangelia.baldimtsi@liu.se; 3 Faculty of Medical Sciences, Örebro University, 701 82 Örebro, Sweden

**Keywords:** biomarkers, diabetic nephropathy, diabetic neuropathy, enzyme-linked immunosorbent assay, glomerular filtration rate, growth differentiation factor 15, high-sensitivity c-reactive protein, matrix metalloproteinase 3, plasma, type 1 diabetes mellitus

## Abstract

Diabetic neuropathy and nephropathy are common complications of type 1 diabetes (T1D). The symptoms are often elusive in the early stages, and available diagnostic methods can be improved using biomarkers. Matrix metalloproteinase 3 (MMP-3) has been identified in the kidneys and is thought to be involved in diabetic nephropathy. Growth differentiation factor 15 (GDF-15) has been suggested to have positive effects in diabetes, but is otherwise associated with adverse effects such as cardiovascular risk, declined kidney function, and neurodegeneration. This study aims to investigate plasma MMP-3 and GDF-15 as systemic biomarkers for diabetic neuropathy and nephropathy in T1D. The study involves patients with childhood-onset T1D (*n* = 48, age 38 ± 4 years) and a healthy control group (*n* = 30, age 38 ± 5 years). Neurophysiology tests, evaluations of albuminuria, and measurements of routine biochemical markers were conducted. The neuropathy impairment assessment (NIA) scoring system, where factors such as loss of sensation and weakened reflexes are evaluated, was used to screen for symptoms of neuropathy. MMP-3 and GDF-15 concentrations were determined in heparinized plasma using ELISA kits. In total, 9 patients (19%) had albuminuria, and 25 (52%) had diabetic neuropathy. No significant differences were found in MMP-3 concentrations between the groups. GDF-15 levels were higher in T1D, with median and interquartile range (IQR) of 358 (242) pg/mL in T1D and 295 (59) in controls (*p* < 0.001). In the merged patient group, a positive correlation was found between MMP-3 and plasma creatinine, a negative correlation was found between MMP-3 and estimated glomerular filtration rate (eGFR; rho = −0.358, *p* = 0.012), and there was a positive correlation between GDF-15 and NIA (rho = 0.723, *p* < 0.001) and high-sensitive C-reactive protein (rho = 0.395, *p* = 0.005). MMP-3 was increased in macroalbuminuria and correlated positively with NIA only in the nine T1D patients with albuminuria (rho = 0.836, *p* = 0.005). The present study indicates that high MMP-3 is associated with low eGFR, high plasma creatinine, and macroalbuminuria, and that GDF-15 can be a biomarker for diabetic neuropathy in T1D. MMP-3 may be useful as biomarker for neuropathy in T1D with albuminuria.

## 1. Introduction

Both neuropathy and nephropathy are frequent complication of type 1 diabetes (T1D). Neuropathy occurs peripherally and affects sensory input. It begins with pain and hyperesthesia, which transitions to hypoesthesia when nerve damage proceeds, sometimes resulting in amputation [1]. Risk factors for developing diabetic neuropathy include age, duration of disease, blood glucose levels, and comorbidities [2]. The symptoms can be elusive at first, making the condition difficult to detect until the disease has advanced [1]. 

The most frequent type of neuropathy around the world is diabetic peripheral neuropathy, with a calculated prevalence of >50% individuals with diabetes [3]. The risk of developing neuropathy appears to increase with the number of years from disease onset. Furthermore, diabetic neuropathy has been found to be challenging to avoid even with close control of blood glucose levels [1]. Currently, there are no dependable methods of curing the condition, and it is treated only by relieving symptoms.

There are several hypotheses regarding the pathology of diabetic neuropathy. Peripheral nerves take up glucose without insulin, making them sensitive to hyperglycemic conditions [1]. Endothelial cells in the blood vessels supplying nerve fibers become damaged, leading to diabetic neuropathy [1]. Another complication of diabetes is diabetic kidney disease, which can cause end-stage renal disease if not correctly treated [4,5,6]. Currently, the urine ratio of albumin and creatinine or the estimated glomerular filtration rate (eGFR) are used for monitoring diabetic kidney disease. However, studies have indicated that these methods are affected by several physiological processes and are not sensitive enough to indicate kidney disease in the early stages. Diabetic nephropathy is considered difficult to cure when it has transitioned to microalbuminuria or macroalbuminuria, and treatment in the early stages of the disease seems to be more effective [7].

Matrix metalloproteinases (MMPs) are endopeptidases which break down and remake proteins such as elastin and collagen [8]. MMPs need zinc and calcium to function, and over 20 types have been found. Different MMPs have different functions, for instance, MMP-1 is a collagenase, MMP-2 and MMP-9 are gelatinases, while MMP-3 and MMP-10 are stromelysines. Their effects are inhibited by tissue inhibitors of metalloproteinases (TIMP 1–4) and α2-macroglobulin [9]. TIMPs act by reversible binding to the catalytic domain of MMPs. They are further thought to be involved in the homeostasis of the extracellular matrix (ECM), and elevated levels of circulating TIMP-1 have been found in patients with metabolic syndrome and diabetes [8]. Different MMPs and TIMPs are suggested as useful biomarkers for diabetic complications, and the balance likely affects pathological conditions, including diabetic neuropathy and nephropathy, in T1D [10,11]. In type 2 diabetes (T2D), MMP breakdown of ECM components in the glomerular basement membrane has been identified as a factor causing nephropathy [12].

MMP-3 is produced by several cell types, including macrophages, endothelial cells, epithelial cells, and fibroblasts [8]. It can degrade collagen, fibronectin, laminin, elastin, and proteoglycans [13]. Moreover, MMP-3 is involved in the activation of other MMPs as well as pro-inflammatory mediators [12,13]. MMP-3 has been found in both the kidney tubules and glomeruli [13]. It has earlier been related to macroalbuminuria and could, therefore, have a role in diabetic nephropathy. MMP-3 breaks down type IV collagen, which is abundant in the glomerular membrane of the kidneys. This is a possible reason for the association with albuminuria [9]. In addition, MMP-3 is involved in inflammation in adipose tissue. However, its role in the human metabolism is not well studied. As MMP-3 is secreted by macrophages in adipose tissue, it is thought to be involved in insulin resistance stimulated by fatty acids [8]. Genetic studies have shown that single-nucleotide polymorphisms (SNPs) in MMP genes give rise to different expressions that may alter the development and progress of diseases such as T2D, cancer, and polycystic kidney disease [12]. Individuals carrying certain genotypes of MMP-3 are thought to be more affected by nephropathy in T2D. 

The cytokine growth differentiation factor 15 (GDF-15) is part of the transforming growth factor β (TGF-β) superfamily and is involved in regulation of multiple systems [14]. GDF-15 can be found in both the central and peripheral nervous systems, where it is mainly produced by the choroid plexus, microglia, injured neurons, and Schwann cells [15]. Secretion of GDF-15 is commonly low in non-reproductive organs, but is raised due to stimuli of hypoxia, tissue injury, and inflammation [16,17]. GDF-15 activates several signaling pathways, including MAPK, PI3K/AKT, and STAT3, and the regulation of GDF-15 involves elements such as p53, reactive oxygen species, as well as TNF-α [18]. GDF-15 has been reported to have positive effects on blood vessels and their cellular function. It is suggested that expression increases when endothelial cells are exposed to high glucose levels and that this effect could be beneficial in diabetes [14]. On the other hand, GDF-15 is associated with inflammation, and increased serum levels have been seen in patients with critical illness and sepsis [14]. In addition, studies have shown that GDF-15 may be involved in the maintenance of cellular respiration, metabolism regulation, and insulin sensitivity [14,16]. GDF-15 has also been reported to exert renal-protective effects [18].

GDF-15 is mostly associated with adverse effects in humans, and individuals with TD2 have been found to have increased levels of circulating GFD-15 [16]. In T2D, elevated levels of circulating GDF-15 are possibly due to increased inflammation [14]. Increased levels of GDF-15 in diabetes are often linked to vascular complications and mortality [17,19], and studies have shown that GDF-15 could be used to identify people at risk of glucose intolerance [17]. Moreover, increased levels of GDF-15 have been reported in individuals with the metabolic syndrome, which correlated to high-sensitivity C-reactive protein (hs-CRP) and elevated glucose levels [20]. Recently, GDF-15 has also been reported to be a predictive marker of neuropathy in T2D [21].

Since it has been indicated that high levels of GDF-15 are found in individuals with neurodegenerative diseases, GDF-15 has potential as a biomarker for diagnosis and prognosis [15]. GDF-15 has also been suggested to predict decline of renal function in diabetic nephropathy [19,20,22]. Studies have similarly shown that measurements of circulating GDF-15 could be used to assess the prognosis and progression of chronic kidney disease [5,6], and that GDF-15 has been associated with the prediction of development of micro- and macroalbuminuria in T2D [7]. GDF-15 has also been associated with diabetic kidney disease in T1D and has shown a correlation with eGFR [19,23].

### Study Aim

The purpose of this study is to examine whether circulating MMP-3 and GDF-15 can be used as biomarkers for detecting and monitoring diabetic neuropathy and/or nephropathy in T1D.

## 2. Results

### 2.1. Precision and Accuracy

For intra-assay precision (Table 1a), three samples for MMP-3 and five samples for GDF-15 were tested individually eight times on the same plate. Six samples were tested in two different plates to determine inter-assay precision (Table 1b) for each analysis method.

The quality controls were analyzed in each test round. Concentrations were within the adequate range for all runs except the low MMP-3 control, which was too low in three out of five runs. These results were 0.722, 0.654, and 0.646 ng/mL. The acceptable range ±3 standard deviations (SD) was 0.756–1.23 ng/mL. Samples were accepted if the intra-assay was ≤5 CV(%), otherwise they were reanalyzed. This was achieved for all samples except for one with MMP-3, which had a CV of 6.2%, but it was included nonetheless.

### 2.2. Clinical Characteristics

Summarized clinical characteristics of the study participants are shown in Table 2. No differences between the groups were found concerning gender, age, body mass index (BMI), hs-CRP, or MMP-3. Systolic and diastolic blood pressure, glucose levels, HbA1c, eGFR, urine albumin/creatinine ratio, NIA, and GDF-15 were higher in the group with T1D compared to the control group (Table 2).

In this cohort, 39 of the patients (81%) had T1D without albuminuria, and 9 (19%) had T1D with albuminuria. Of the nine patients with albuminuria, five (10% had microalbuminuria, and four (8%) had macroalbuminuria. Furthermore, 23 (48%) of the 48 T1D patients did not have diabetic neuropathy, while 25 (52%) had neuropathy.

In subgroups divided based on albuminuria, there were no significant differences between MMP-3’s median and interquartile range (IQR) concentration in the group without albuminuria of 17.2 (12.9) ng/mL, the mean ± SD of the albuminuria group of 23.1 ± 13.4 ng/mL, and the median (IQR) concentration in healthy controls of 13.2 (13.8) ng/mL. 

When comparing concentrations of GDF-15, the group without albuminuria had a median (IQR) plasma level of 348.9 (162.4) pg/mL, and the group with albuminuria measured 631.8 (711.3) pg/mL. Both of these levels were higher than the mean ± SD of the level in the control group, which was 294.7 ± 58.9 pg/mL (*p* = 0.002; *p* < 0.001). In addition, the group with albuminuria had higher concentrations than the group without albuminuria (*p* = 0.020).

In subgroups based on neuropathy, there were no significant differences in MMP-3 median (IQR) plasma concentrations between the T1D group without neuropathy, which was 17.0 (8.2) ng/mL; T1D with neuropathy, which was 19.3 (15.6) ng/mL; and the control group, which was 13.2(13.8) ng/mL. The GDF-15 concentration was higher in the group with neuropathy, 459.3 (428.2), as compared to the group without neuropathy, 325.2 (103.9) pg/mL (*p* = 0.009).

When comparing MMP-3 concentrations between the genders, males had higher median (IQR) concentrations (21.2 (8.9) ng/mL) compared to women (9.3 (5.9) ng/mL) (*p* < 0.001). This difference was similar in both T1D patients and controls. For GDF-15, there were no significant differences in median (IQR) concentrations between males (330.8 (132.8) pg/mL), and females (337.8 (110.2) pg/mL) (*p* = 0.411).

### 2.3. Correlations

**Figure 1:**
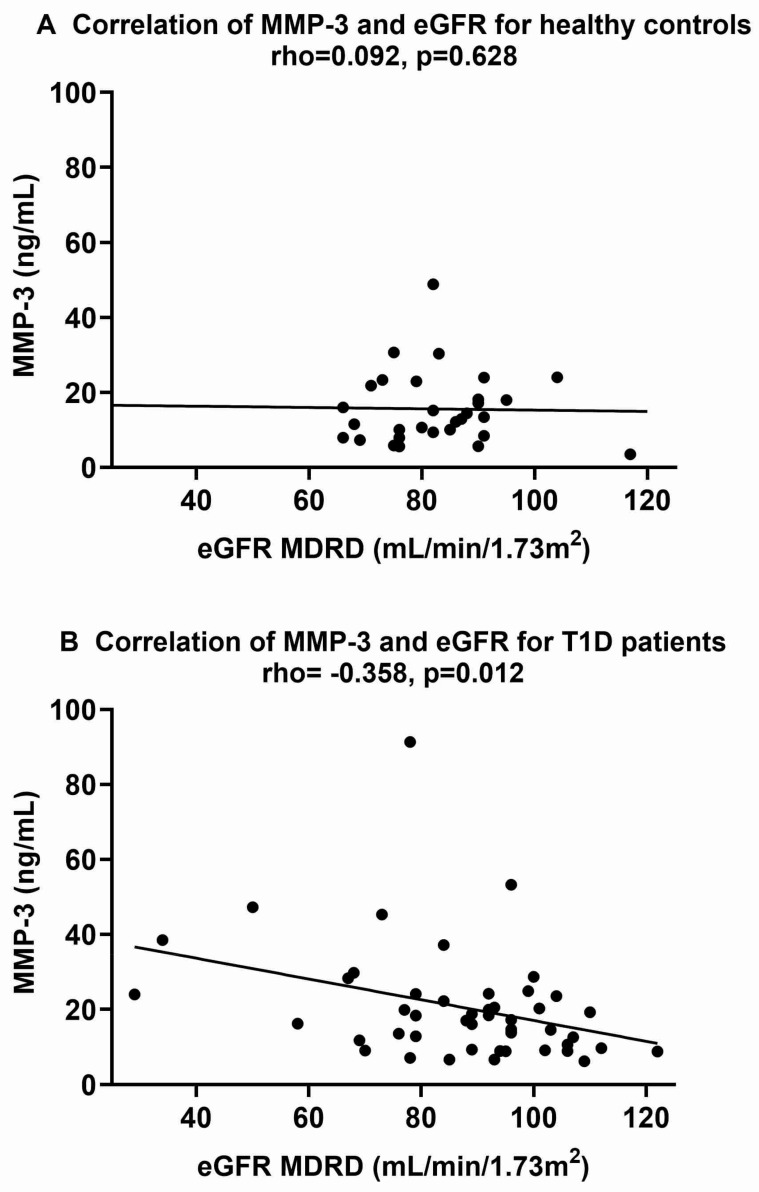
Correlations of plasma MMP-3 with eGFR for healthy controls ((**A**), *n* = 30) and type 1 diabetes (T1D) patients ((**B**), *n* = 48). Spearman’s correlation coefficient was used to assess the relationships. *p* ≤ 0.05 was considered statistically significant. eGFR, estimated glomerular filtration rate; MDRD, Modification of Diet in Renal Disease (MDRD); MMP-3, matrix metalloproteinase 3.

The correlations of plasma concentrations of MMP-3 and GDF-15 in the T1D patients and the controls are shown in Table 3. Significant positive correlations were found between MMP-3 and plasma creatinine and systolic blood pressure in the control group as well as in T1D. In T1D, but not in the controls, a negative correlation was also found between MMP-3 and eGFR (Figure 1). According to local guidelines, the reference interval for eGFR for individuals between the ages of 18 and 70 years is >60 mL/min/1.73 m^2^. Three of the T1D patients had levels below these values (29–58 mL/min/1.73 m^2^). The local reference interval for plasma creatinine is 45–90 µmol/L for women and 60–105 for males. One female T1D patient had 144 µmol/L in plasma creatinine, and two male patients had 121 and 220 µmol/L. The two patients with the highest plasma creatinine did also suffer from macroalbuminuria. 

When analyzing correlations between T1D patients and healthy controls divided by gender, only males showed a positive correlation between MMP-3 and systolic blood pressure (rho = 0.342, *p* = 0.027).

For GDF-15 in the control group, a negative correlation with eGFR was found. Positive correlations were found in the T1D group for GDF-15 with age, hs-CRP, and NIA (Table 3).

### 2.4. Diabetic Nephropathy

When dividing into subgroups, correlations were found between the plasma concentrations of MMP-3 and GDF-15 in the T1D group without albuminuria (*n* = 39), as well as in the T1D group with albuminuria (*n* = 9). In the T1D group without albuminuria, positive correlations were found between MMP-3 and BMI (rho = 321, *p* = 0.046), plasma creatinine (rho = 0.512, *p* = <0.001), and systolic blood pressure (rho = 0.411, *p* = 0.009). MMP-3 was also negatively correlated with HbA1c in this subgroup (rho = −0.427, *p* = 0.007). For GDF-15, positive correlations were found with age (rho = 0.435, *p* = 0.006), hs-CRP (rho = 0.322, *p* = 0.046), and NIA (rho = 0.669, *p* = <0.001). 

**Figure 2:**
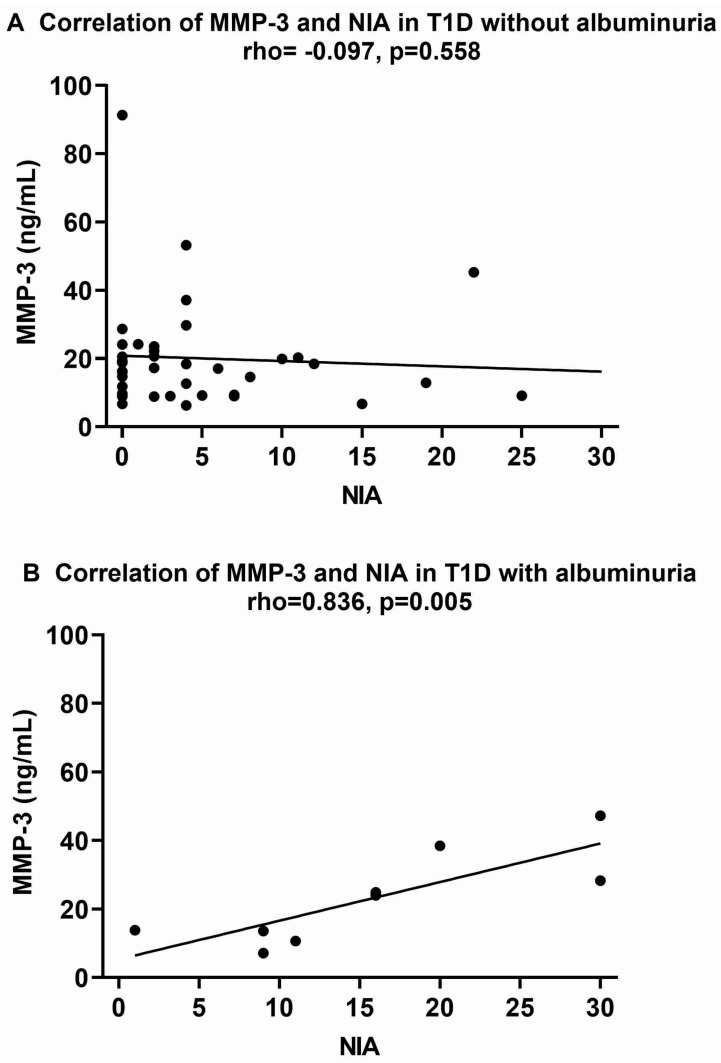
Correlations of plasma MMP-3 with NIA for type 1 diabetes (T1D) patients without albuminuria ((**A**), *n* = 39) and T1D patients with albuminuria ((**B**), *n* = 9). Spearman’s correlation coefficient (rho) was used to assess the relationships. *p* ≤ 0.05 was considered statistically significant. NIA, neuropathy impairment assessment.

For T1D with albuminuria, MMP-3 correlated only with NIA (rho = 0.836, *p* = 0.005, Figure 2). MMP-3 levels were also significantly higher (*p* = 0.032) in four patients with macroalbuminuria, with a median (IQR) of 33.3 (19.9), as compared to five patients with microalbuminuria, with levels of 13.5 (10.5), *n* = 5. GDF-15 was correlated with HbA1c (rho = 0.667, *p* = 0.050), hs-CRP (rho = 0.683, *p* = 0.042), and NIA (rho = 0.684, *p* = 0.042). 

### 2.5. Diabetic Neuropathy

NIA was correlated with GDF-15 in the merged T1D group (Table 3), as well as in the subgroups divided based on neuropathy (Table 4). GDF-15 was correlated with age only in the group without neuropathy. For MMP-3, a positive correlation was found only in the T1D group with neuropathy for systolic blood pressure. 

**Figure 3:**
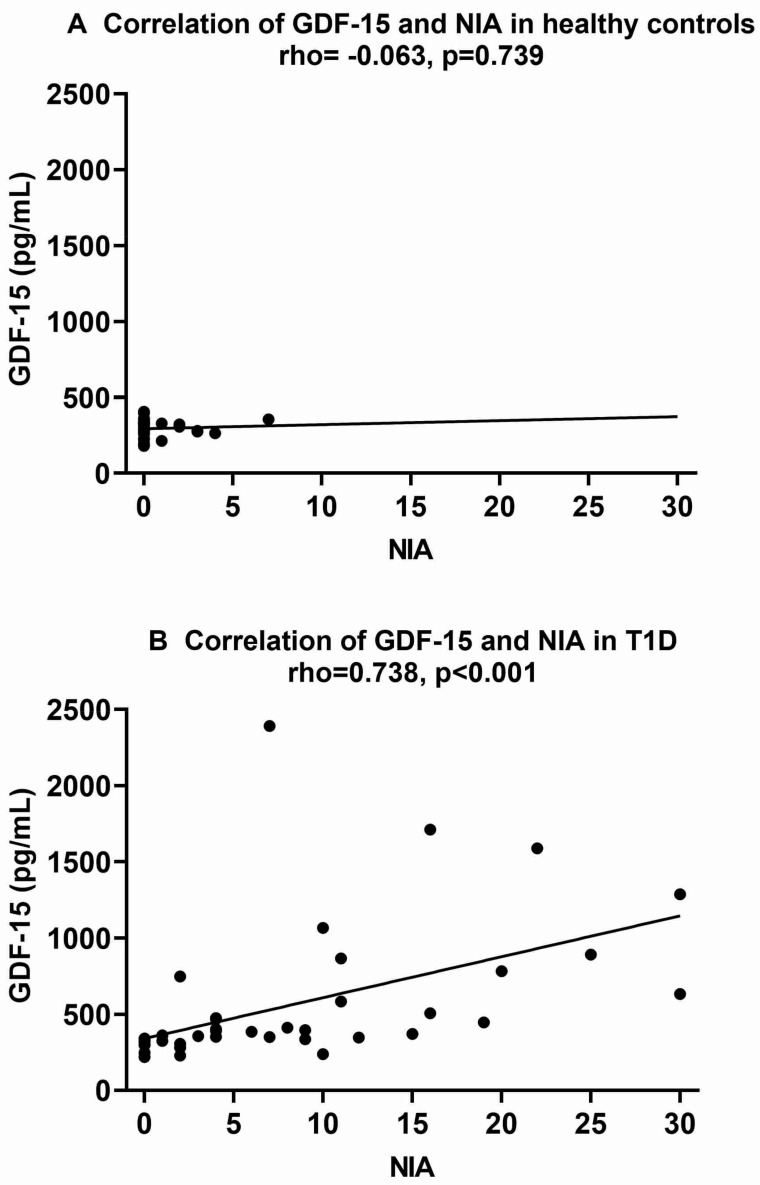
Correlations of plasma GDF-15 with NIA for healthy controls ((**A**), *n* = 30) and type 1 diabetes (T1D) patients ((**B**), *n* = 47). Spearman’s correlation coefficient (rho) was used to assess the relationships. *p* ≤ 0.05 was considered statistically significant. GDF-15, growth differentiation factor 15; NIA, neuropathy impairment assessment.

Plots of the correlations of GDF-15 and NIA are shown in Figure 3. In Figure 3B, an outlier from a T1D patient with a GDF-15 concentration of 9827 pg/mL is excluded. The effects of this exclusion were rho = 0.738, *p* < 0.001 without the outlier compared to rho = 0.723, *p* < 0.001 with the outlier.

When comparing concentrations of hs-CRP in the neuropathy subgroups, no significant difference was found between the median (IQR) concentrations in T1D without neuropathy (0.80 (1.70) mg/mL) and healthy controls (0.70 (1.43) mg/mL). However, the T1D group with diabetic neuropathy had a higher median (IQR) concentrations of 3.00 (3.50) mg/mL compared to both T1D without neuropathy (*p* = 0.030) and the control group (*p* = 0.035).

## 3. Discussion

In this study, the intention was to investigate the potential of MMP-3 and GDF-15 as biomarkers for neuropathy and nephropathy in T1D. The findings indicate that plasma GDF-15 may be a prognostic marker for diabetic neuropathy due to its association with NIA. These results are consistent with a recent study including 241 T2D patients, which suggested that serum GDF-15 is a predictive marker of neuropathy [21]. It has previously been shown that GDF-15 increases due to several factors, including tissue injury and inflammation [16,17], and that it is possibly involved in the development of T2D [16]. Even though elevated GDF-15 levels have been suggested to have beneficial effects in diabetes [14], they have also been linked to increased mortality [17,19]. In the present study, an association of GDF-15 and NIA was found in all T1D groups.

This study showed that the GDF-15 concentrations were approximately 20% higher in the T1D group compared to healthy controls, and that subgroups with complications (albuminuria and neuropathy) had the highest concentrations. GDF-15 has been reported as an early biomarker of kidney disease [18]. The present study showed that GDF-15 correlates negatively with eGFR only in healthy controls. GDF-15 did not correlate with any marker of renal function in any group of T1D patients, and GDF-15 showed no association with nephropathy. However, the present study only included nine patients with albuminuria, and associations of GDF-15 with other parameters of renal function, such as creatinine clearance, were not investigated. No significant association with gender was found for GDF-15, in accordance with a recent report [24], but conflicting results have been found in studies of T2D [25]. In contrast to [24], no associations between GDF-15 and BMI were found in this study, but the levels of GDF-15 in T1D patients correlated with age in accordance with the same study.

Furthermore, correlations of GDF-15 and hs-CRP were found in the whole T1D group, but not in any neuropathy subgroup. Elevated GDF-15 levels have previously been related to hs-CRP in individuals ≥ 65 years of age with metabolic syndrome [20]. No difference in hs-CRP was found comparing T1D and healthy controls in the present study, but the neuropathy subgroup had concentrations 3.2 times higher than the T1D group without neuropathy and 2.9 times higher than the control group. T1D is a disease with chronic immunoinflammation, which can explain the increased hs-CRP concentrations in the patient group with neuropathy [26]. In the correlation plots, one patient with an hs-CRP concentration above the normal reference (33 mg/L, reference < 10 mg/L) was excluded due to the possibility of this patient having an ongoing infection or acute inflammatory reaction affecting the measured biomarkers. The balance of MMPs and their inhibitors, TIMPs, is suggested to impact nephropathy [11], and increased concentrations of TIMP-1 are associated with diabetes [8]. The relationship between MMP-3 and gender was identified in several groups and subgroups in the present study, and the MMP-3 concentrations in males were found to be 2.3 times higher compared to women, which is consistent with previous findings [27]. 

MMP-3 determined in serum has been related to macroalbuminuria in T1D [9], and this is in accordance with the present study’s findings of increased plasma MMP-3 in T1D with macroalbuminuria. Then again, the number of patients with macroalbuminuria in this cohort is limited (*n* = 4), and the total number of patients with nephropathy is small (*n* = 9), meaning that interpretations need to be made with caution. Furthermore, higher plasma MMP-3 was associated with higher plasma creatinine and lower eGFR, as has been reported earlier [13], and a recent study suggested that MMP-3 levels in serum could assist eGFR in the diagnosis of early chronic kidney disease [28]. TIMP-1 and neutrofil gelatinase-associated lipocalin (NGAL) are other markers that have also been reported to negatively correlate with eGFR in T1D [11].

Intra-assay analyses of MMP-3 showed better results than the number specified by the production company. On the other hand, more variation was found in the inter-assay analysis compared to the coefficients provided by R&D Systems. Nota bene: fewer numbers of repetitions and separate assays were used in this precision testing. The low level of the MMP-3 control was below the acceptable range in three out of five runs. This could indicate that the analysis kit is less reliable at low MMP-3 levels. For GDF-15, the quality control was in the acceptable range in all runs. However, the sample concentration of GDF-15 for one of the patients was vastly higher than all other samples and nearly ten times higher than the maximum point in the standard curve, and therefore had to be diluted further, rendering the result less reliable. This is a reason for removing it as an outlier in the correlation plots.

The strengths of this study are that the T1D group was homogenous and unselected from a limited geographic area. All patients were diagnosed with T1D before 15 years of age, and they were continuously monitored. The control group with individuals similar in terms of age and gender had the same exclusion criteria as the patient group and underwent the same examinations.

This study has several limitations. The size and composition of the patient cohort is limited by the fact that it consists of subjects with childhood-onset T1D who have been followed prospectively. When sub-grouping the patient group, the size of the material was reduced resulting in reduced statistical power, which may have led to associations remaining undetected, as well as a risk of bias. Furthermore, the study is cross-sectional, and samples for biomarker analysis were collected at a single point in time. Some biomarkers varied in concentration over the day or month, and no follow-ups was made. One potential outlier for GDF-15 was removed in the correlation plot, but the removal did not notably affect the correlation coefficient. All measurements were used in the other analyses. Neuropathy is probably affected by several factors, including age, BMI, and different MMPs and TIMPs. T1D and/or the complications, in turn, likely change factors such as CRP and biomarkers such as MMPs and TIMPs. All of this taken together, the relationship between both GDF-15 and MMP-3 with complications of T1D must be investigated in further studies.

In conclusion, this study has shown promising results regarding MMP-3 and GDF-15 as biomarkers for diabetic nephropathy and neuropathy. MMP-3 could be valuable as a complement to other analyses to assess kidney damage. Strong associations between high GDF-15 concentrations and high NIA scores were found in all investigated patient groups. Diabetic neuropathy is a frequent complication of T1D; it is difficult to avoid, and no definite treatment is available. Identifying patients at risk is therefore crucial in order to minimize risk factors and further complications. This is, to our knowledge, the first study identifying the association between GDF-15 and NIA in T1D, and due to the limited number of patients studied, this association should be investigated further.

## 4. Materials and Methods

### 4.1. Patients

In this analysis, data and plasma from a cross-sectional study earlier described by Baldimtsi et al., 2023 [10] were used. Inclusion was performed at the Department of Endocrinology at the University Hospital of Linköping. The study recruited 48 individuals diagnosed with T1D in childhood (20 female, 28 male), aged between 31 and 46 years, with a mean age ± standard deviation (SD) of 38.4 ± 3.9 years. The average disease duration at the time of data collection was 30.7 ± 5.3 years.

Inclusion criteria were defined as a diagnosis of T1D with intensive insulin therapy, no history of other metabolic or neurological diseases, no use of pharmacological treatments known to affect the nervous system, and no history of alcohol abuse. The diagnosis of T1D was based on the presence of autoantibodies against a minimum of one of the following: glutamic acid decarboxylase (GADA), islet antigen 2 (IA2A), or islet cells (ICA). 

### 4.2. Healthy Controls

The control group involved 30 healthy volunteers (16 female, 14 male) aged 29 to 48 years, with a mean age of 37.9 ± 5.5 years. Exclusion criteria were genetic neurological disease, metabolic disorders, drug and alcohol abuse, and medical treatment interfering with nerve function.

### 4.3. Nephrology and Neurology Assessment

Albuminuria was used to evaluate diabetic nephropathy. All test results of urine albumin < 3 mg/L were noted as 2 mg/L for the calculation of the albumin/creatinine ratio. The definitions of microalbuminuria used were two urine test results in the same year with either an albumin/creatinine ratio of 3–30 mg/mmol or a rate of excretion of albumin of 20–200 µg/min. For macroalbuminuria, the albumin/creatinine ratio was defined as >30 mg/mmol, or in the case of the excretion rate of albumin, >200 µg/min. Calculations of eGFR were made using the Modification of Diet in Renal Disease (MDRD) formula [29].

Neurophysiology was tested using motor nerve conduction velocity, sensory nerve conduction velocity, and compound muscle action potential, as described by Papadopoulou-Marketou et al., 2021 [11]. To define neuropathy, the Toronto criteria using a manifestation of an abnormal nerve conducting result and a sign of neuropathy were used [30]. If no signs of neuropathy were found, but test results of nerve conduction were abnormal, it was defined as subclinical diabetic neuropathy. Furthermore, neuropathy impairment assessment (NIA) was used to screen participants for symptoms of neuropathy [31]. NIA is a scoring system evaluating touch, pinprick, vibration, and temperature perceptions. A high score indicates more severe neuropathic damage.

### 4.4. Biochemical Markers 

Blood samples were collected after 10 h of fasting. Routine biochemical markers (P-glucose, B-HbA1c, P-hs-CRP, P-creatinine, U-creatinine, and U-albumin) were analyzed at the accredited Department of Clinical Chemistry at the University Hospital of Linköping, Sweden. For the measurement of MMP-3 and GDF-15, heparinized blood was collected and centrifuged at 1500× *g* for 15 min before the plasma was aliquoted and frozen at −80 °C. The aliquots were stored at the Linköping Biobank Facility, University Hospital, Sweden. Levels of MMP-3 and GDF-15 were measured at the Division of Clinical Chemistry and Pharmacology, Linköping University, Sweden, using Quantikine ELISA kits provided by R&D Systems (Minneapolis, MN, USA).

MMP-3 was analyzed using the Quantikine ELISA kit. Plasma samples were diluted 10-fold before analysis. Samples were incubated with diluent for 2 h at room temperature on a microplate shaker at 500 rpm. Wells were then washed manually four times before the conjugate solution was added. The plate was then placed on the shaker again for 2 h at room temperature. The wells were washed as before, and substrate solution was added. The plate was incubated for 30 min at room temperature, protected from light. Stop solution was then added to each well, and optical density was determined using a microplate reader set to 450 nm, with wavelength correction set to 540 nm. Readings were made within 30 min of adding the stop solution. A standard curve was generated using a log/log curve fit. Indicated inter-assay coefficients of variation were between 7.0% and 8.6%, and the intra-assay coefficients were 5.7% and 6.4% (R&D Systems).

For the analysis of GDF-15, the Quantikine QuickKit ELISA was used. A 2-fold dilution was made for all plasma samples before analysis. Samples were then incubated with the antibody cocktail on a microplate shaker set at 500 rpm for 1 h at room temperature. The wells were washed manually three times before the addition of substrate solution. The plate was then incubated for 20 min at room temperature, protected from light. The stop solution was added, and optical density was determined within 30 min using a microplate reader set to 450 nm with wavelength correction at 540 nm. A standard curve was created using a four-parameter logistic (4-PL) curve fit. Provided by the company, the inter-assay coefficients of variation were between 5.7% and 8.4%, and the intra-assay coefficients were 3.9% and 6.7%. 

Each sample was analyzed in duplicate and reanalyzed if intra-assay CV exceeded 5%. Quantitative controls provided by R&D Systems (Minneapolis, MN, US) were used in each test round. The “Quantikine ELISA Kit Control Set 637”, customized for human MMP-3, was used in three levels with the following acceptable ranges (±3 SD): low (0.756–1.23 ng/mL), mid (2.36–3.85 ng/mL), and high (4.52–7.37 ng/mL). For GDF-15, the “Quantikine QuickKit Immunoassay Control Group 282” for human GDF-15 was used at two concentrations with the following acceptable ranges (±3 SD): low (113–209 pg/mL) and high (654–1214 pg/mL). Each vial was reconstructed with deionized water, aliquoted, and frozen at −20 °C.

### 4.5. Ethical Considerations

Informed consent to take part in the study, with the possibility to withdraw from continued involvement without further explanation, was obtained from all participants. The plasma samples and collected test results used in this study were pseudonymized. The regional ethical committee in Linköping approved the study (Dnr: 2017/190–31).

### 4.6. Statistical Analysis

Statistical analyses were performed using the software SPSS version 28 (IBM Corporation, Armonk, NY, USA). Statistical significance was considered at *p* ≤ 0.05.

The Shapiro–Wilk test was used to analyze the normality of the distribution of the variables. Continuous variables are presented as mean (±SD) if normally distributed or median and interquartile range (IQR) if non-normally distributed. Categorical variables are shown as frequencies (relative frequencies). Independent-sample *T*-tests for normally distributed variables and Mann–Whitney *U*-tests for non-normally distributed variables were used to determine differences between two groups. For comparisons between more than two groups, the Kruskal–Wallis test was applied. For bivariate correlation analyses, Spearman’s rank correlation (rho) was used, except when both variables were normally distributed, in which case the Pearson correlation (r) was utilized.

The sample size provided an estimated statistical power of 78% in the T1D patients (*n* = 48) and 56% power in the control group (*n* = 30) for a test of the null hypothesis that the Spearman correlation (two-sided) between different parameters was zero versus the alternative hypothesis that the correlation (rho) was greater than or equal to 0.4, or less than or equal to −0.4. Power analysis was based on Fisher’s z-transformation and normal approximation. Variance was estimated by the method proposed by Bonett and Wright.

## Figures and Tables

**Table 1:** Intra-assay (a) and inter-assay (b) precision for MMP-3 and GDF-15 using the Quantikine ELISA kit and the Quantikine QuickKit ELISA from R&D Systems (Minneapolis, MN, USA), respectively.

a	MMP-3 (ng/mL)							GDF-15 (pg/mL)											
Sample	1		2		3			1			2		3			4		5	
n	8		8		8			8			8		8			8		8	
Mean	5.26		28.1		8.76			907.8			381.8		637.3			520.1		235.8	
Standard deviation	0.027		0.077		0.030.			9.83			5.38		10.3			8.25		2.93	
CV (%)	5.2		2.7		3.4			2.2			2.8		3.2			3.2		2.5	
**b**	**MMP-3 (ng/mL)**									**GDF-15 (pg/mL)**									
Sample	1	2		3		4	5		6	1		2		3	4		5		6
n	2	2		2		2	2		2	2		2		2	2		2		2
Mean	23.9	36.9		24.9		23.9	8.62		10.4	896.1		352.3		544.2	366.9		492.3		213.1
Standard deviation	0.02	1.57		1.42		1.74	0.33		0.27	4.43		19.6		39.4	3.29		14.3		13.1
CV (%)	0.1	6.0		8.1		10	5.4		3.7	0.7		7.8		10	1.3		4.1		8.7

MMP-3, matrix metalloproteinase 3; GDF-15, growth differentiation factor 15.

**Table 2:** Study participants’ characteristics, divided into patients with type 1 diabetes (T1D) and healthy controls.

Parameter	T1D			Controls			*p*-Value
N	48 *			30 **			
Male	28 (58.3%)			14 (46.7%)			0.318
	M	Min	Max	M	Min	Max	
Age (years)	38.4 (±3.9)	31	46	37.9 (±5.5)	29	48	0.690
Diabetes duration (years)	30.7 (±5.3)	20	43	-	-	-	-
Body mass index (kg/m^2^)	26.0 (4.5)	21.1	37.9	25.4 (6.0)	19.0	45.8	0.385
Systolic blood pressure (mmHg)	131.5 (16.8)	106	179	113.4 (±13.7)	86.0	140	**<0.001**
Diastolic blood pressure (mmHg)	82.4 (±11.0)	63.0	112	72.4 (±8.14)	52.0	91.0	**<0.001**
P-Glucose (mmol/L)	12.3 (6.6)	1.8	31.0	5.4 (±0.4)	4.5	6.1	**<0.001**
B-HbA1c (mmol/mol)	59.9 (±14.5)	27.0	100.0	32.4 (±3.1)	26.0	39.0	**<0.001**
P-Creatinine	80.4 (28.2)	43.0	220.0	76.2 (±12.5)	52.0	100.0	0.433
eGFR MDRD(mL/min/1.73 m^2^)	91.5 (22.0)	29	122	82.8 (±11.3)	66	117	**0.031**
U-albumin/creatinine ratio (mg/mmol)	0.56 (0.83)	0.03	112.00	0.28 (0.16)	0.14	2.34	**<0.001**
P-hs-CRP (mg/mL)	1.6 (2.7)	0.2	33.0	0.7 (1.4)	0.2	6.3	0.247
NIA	4 (11)	0	30	0 (1)	0	7	**<0.001**
P-MMP-3 (ng/mL)	17.8 (14.1)	6.2	91.3	13.2 (13.8)	3.5	48.8	0.090
P-GDF-15 (pg/mL)	358.5 (242.3)	214.3	9827	294.7 (±58.9)	178.5	406.9	**<0.001**

P, plasma; B, blood; U, urine; eGFR, estimated glomerular filtration rate; MDRD, estimated glomerular filtration rate; hs-CRP, high-sensitivity C-reactive protein; NIA, neuropathy impairment assessment; MMP-3, matrix metalloproteinase 3; GDF-15, growth differentiation factor 15. Results are presented as counts and percentages, mean ± standard deviation (±SD), or median and interquartile range (IQR), with minimum and maximum values. *p*-value, statistical significance (*p* ≤ 0.05) between T1D and controls. * For glucose, *n* = 47, and for albumin/creatinine ratio, *n* = 42. ** For albumin/creatinine ratio, *n* = 26.

**Table 3:** Correlations of plasma MMP-3 and GDF-15 in the control group and the type 1 diabetes (T1D) group.

Groups		P-MMP-3 (ng/mL)		P-GDF-15 (pg/mL)	
		rho	*p*-Value	rho/r	*p*-Value
**Controls** N = 30 *	Age (years)	−0.229	0.224	0.111 #	0.599
	Body mass index (kg/m^2^)	−0.164	0.387	−0.091	0.633
	Systolic BP (mmHg)	**0.403**	**0.027**	−0.203 #	0.283
	Diastolic BP (mmHg)	0.244	0.194	−0.120 #	0.529
	P-Glucose (mmol/L)	0.150	0.429	−0.036 #	0.849
	B-HbA1c (mmol/mol)	0.199	0.530	−0.009 #	0.963
	P-Creatinine	**0.632**	**<0.001**	0.100 #	0.599
	eGFR (mL/min/1.73 m^2^)	0.092	0.628	**−0.622** #	**<0.001**
	U-albumin/creatinine ratio (mg/mmol)	0.034	0.870	0.031	0.880
	P-hs-CRP (mg/mL)	−0.152	0.423	0.019	0.919
	NIA	0.004	0.982	−0.063	0.739
**T1D ** N = 48 **	Diabetes duration (years)	0.021	0.888	0.167	0.258
	Age (years)	0.159	0.279	**0.395**	**0.005**
	Body mass index (kg/m^2^)	0.240	0.100	−0.020	0.895
	Systolic BP (mmHg)	**0.502**	**<0.001**	−0.014	0.926
	Diastolic BP (mmHg)	0.216	0.141	−0.014	0.926
	P-Glucose (mmol/L)	0.141	0.345	−0.045	0.765
	B-HbA1c (mmol/mol)	−0.253	0.082	0.240	0.100
	P-Creatinine	**0.514**	**<0.001**	−0.016	0.916
	eGFR (mL/min/1.73 m^2^)	**−0.358**	**0.012**	−0.259	0.075
	U-albumin/creatinine (mg/mmol)	0.046	0.774	0.176	0.264
	P-hs-CRP (mg/mL)	0.004	0.977	**0.395**	**0.005**
	NIA	0.094	0.524	**0.723**	**<0.001**

BP, blood pressure; P, plasma; B, blood; U, urine; eGFR, estimated glomerular filtration rate; hs-CRP, high-sensitivity C-reactive protein; MMP-3, matrix metalloproteinase 3; GDF-15, growth differentiation factor 15; NIA, neuropathy impairment assessment. Statistical significance (*p* ≤ 0.05) marked in bold. Analyses marked with # are presented with Pearsons’s correlation coefficient (r); all other correlations are presented with Spearman’s correlation coefficient (rho). * For albumin/creatinine ratio, *n* = 26. ** For glucose, *n* = 47, and for albumin/creatinine ratio, *n* = 42.

**Table 4:** Correlations of plasma MMP-3 and GDF-15 in type 1 diabetes (T1D) subgroups based on neuropathy.

Subgroups		MMP-3 (ng/mL)		GDF-15 (pg/mL)	
		rho	*p*-Value	rho	*p*-Value
**T1D without neuropathy ** N = 23 *	Age (years)	0.165	0.451	0.407	0.050
	Diabetes duration (years)	0.045	0.839	0.206	0.345
	Body mass index (kg/m^2^)	0.296	0.170	−0.087	0.691
	Systolic BP (mmHg)	**0.525**	**0.010**	−0.179	0.413
	Diastolic BP (mmHg)	0.192	0.381	−0.259	0.232
	P-Glucose (mmol/L)	0.340	0.113	−0.405	0.055
	B-HbA1c (mmol/mol)	0.024	0.914	−0.143	0.514
	P-Creatinine	**0.566**	**0.005**	0.160	0.466
	eGFR (mL/min/1.73 m^2^)	**−0.415**	**0.049**	−0.293	0.176
	U-albumin/creatinine ratio (mg/mmol)	−0.304	0.271	−0.396	0.143
	P-hs-CRP (mg/mL)	−0.103	0.639	0.299	0.166
	NIA	−0.054	0.808	**0.589**	**0.003**
**T1D with neuropathy ** **N = 25** **^¥^**	Age (years)	0.165	0.430	0.380	0.061
	Diabetes duration (years)	0.004	0.985	0.018	0.931
	Body mass index (kg/m^2^)	0.162	0.440	−0.025	0.904
	Systolic BP (mmHg)	**0.508**	**0.010**	0.020	0.923
	Diastolic BP (mmHg)	0.258	0.214	0.063	0.766
	P-Glucose (mmol/L)	−0.033	0.878	0.092	0.670
	B-HbA1c (mmol/mol)	−0.359	0.078	0.364	0.074
	P-Creatinine	**0.436**	**0.029**	0.118	0.574
	eGFR (mL/min/1.73 m^2^)	−0.325	0.113	−0.239	0.249
	U-albumin/creatinine ratio (mg/mmol)	0.127	0.616	0.442	0.066
	P-hs-CRP (mg/mL)	−0.019	0.927	0.307	0.135
	NIA	0.277	0.180	**0.674**	**<0.001**

BP, blood pressure; P, plasma; B, blood; U, urine; eGFR, estimated glomerular filtration rate; hs-CRP, high-sensitivity C-reactive protein; MMP-3, matrix metalloproteinase 3; GDF-15, growth differentiation factor 15; NIA, neuropathy impairment assessment. Correlations are presented with Spearman’s correlation coefficient (rho). Statistical significance (*p* ≤ 0.05) marked in bold. * For albumin/creatinine ratio, *n* = 20. ^¥^ For glucose, *n* = 25, and for albumin/creatinine ratio, *n* = 22.

## Data Availability

The data presented in this study are available upon reasonable request from the corresponding author.
